# Two-Step Measurement of Water–Energy–Food Symbiotic Coordination and Identification of Key Influencing Factors in the Yangtze River Basin

**DOI:** 10.3390/e23070798

**Published:** 2021-06-23

**Authors:** Weizhong Chen, Yan Chen

**Affiliations:** 1 College of Economics and Management, Nanjing Forestry University, Nanjing 210037, China; chenweizhong0713@163.com; 2 Research Institute of Ecological Civilization Construction and Forestry Development with Chinese Characteristics, Nanjing Forestry University, Nanjing 210037, China

**Keywords:** BP-DEMATEL-GTCW model, game theory combination weight, key influencing factors identification, Lotka–Volterra model, water–energy–food symbiotic coordination, Yangtze River Basin

## Abstract

With the intensification of people’s production and life behaviors, the systemic risks of water, energy and food in the Yangtze River Basin have become increasingly prominent, which has become a bottleneck for sustainable development of social, economic and ecological in the basin. Therefore, studying the symbiotic coordination between water, energy and food is of great significance to promoting regional sustainable development. First, from the perspective of water–energy–food symbiosis, with the water–energy–food ecosystem conceptual model as the nexus, the two-step measurement model of the symbiotic index and the symbiotic level index is used to study the water–energy–food symbiosis of the Yangtze River. Then, we use the BP-DEMATEL-GTCW model to identify the key influencing factors that affect the symbiotic security of the water–energy–food ecosystem. In this research, it is found that the average value of the symbiotic degree of the water–energy–food ecosystem of the 11 provinces or municipalities in the Yangtze River Basin only reached the risk grade. It can also be seen from the identification results of key influencing factors that energy microsystem-related indicators have a greater impact on the symbiotic development of the entire WEF ecosystem. Therefore, special attention needs to be paid to increasing energy sources and reducing expenditure. Relevant departments need to effectively develop primary energy production and expand energy-saving investment through multiple channels to expand energy self-sufficiency and ultimately promote the coordinated and effective development of water, energy and food in the Yangtze River Basin.

## 1. Introduction

As strategic basic resources, water, energy and food are the most important resources needed for human survival and development. They not only meet the basic needs of human production and life, but also play an important role in environmental protection. The three resources of water, energy and food are interdependent and closely related. Specifically, the process of energy extraction, processing and transformation requires water, and the purification, distribution and dispatch of water also require energy. Furthermore, the irrigation, growth, harvest, transportation and other production or utilization links of food are inseparable from the consumption of water resources and energy. At present, freshwater resources are increasingly scarce, food supply uncertainties are increasing, and social demand for energy is increasing. The three resources have new features of interdependence and conflict, making the bond between water, energy and food even more complicated. However, human progress and development are still facing three main problems: population expansion, resource shortage and environmental degradation. These problems are closely related to the water–energy–food system [1]. With population growth and socio-economic development, the systemic risks of water, energy and food have become increasingly prominent, and have attracted great attention from governments and academic circles. Therefore, studying the symbiotic coordination between water, energy and food is of great significance to promoting regional sustainable development.

Since the water–energy–food nexus was first emphasized at the Bonn 2011 conference, many scholars have begun to study it from the perspectives of theory and practice. Additionally, some research studies have been widely recognized by institutions in related fields and academia [2,3,4,5]. Some scholars start from the internal relationship of the water–energy–food nexus and explain how to better understand it [6]. In order to show its internal relationship more clearly, many scholars use a variety of diagrams to show the water–energy–food nexus [7,8,9,10]. Moreover, there will be certain challenges in the development of the relationship between water, energy and food [11,12], and some measures will help to meet the challenge [13,14,15]. On the basis of the above qualitative analysis, academia has begun to assess water–energy–food nexus. At present, the research of water–energy–food has developed to the stage of combining theoretical research with practical research. In terms of qualitative theoretical research, most of the current articles assess water–energy–food from the perspectives of security, resilience, sustainability, and synergy. Research from the perspective of security mainly analyzes the security pattern of the water–energy–food nexus, objectively evaluates its security, and better proposes system security strategies to ensure its system security [16,17,18]; Research from the perspective of resilience mainly analyzes the ability of the water–energy–food system to recover under self-organization and external forces when it is damaged by external forces, and formulates related strategies to improve the resilience of the water–energy–food system [19,20,21]; The research from the perspective of sustainability mainly analyzes and evaluates the sustainability of the water–energy–food system, and aims to promote sustainable development that takes into account random and risk factors, so as to better enable it to maintain a healthy state of development for a long time [22,23,24,25]; The research from the perspective of synergy mainly studies the coordinated, cooperative or synchronized joint effects and collective behaviors of the internal subsystems of the water–energy–food system, whose purpose is to coordinate their internal relationships. Moreover, these research studies are of great significance to realize the harmonious development of regional water, energy and food [26,27,28]. In terms of quantitative practical research, there are currently many specialized or interdisciplinary methods used in the research of water–energy–food. Common quantitative methods include: WEF nexus tool 2.0 [29], life cycle assessment (LCA) [30,31], computable general equilibrium model (CGE) [32], System dynamics model (SD) [33,34,35], climate, land, energy and water strategies (CLEWS) [36,37], multi-scale integrated analysis of societal and ecosystem metabolism (MuSIASEM) [38,39], water evaluation and planning–long-range energy alternatives planning system (WEAP-LEAP) [40], nexus simulation system (NexSym) [41]. These methods quantify and evaluate the relationship between water, energy, and food from the perspective of the water–energy–food nexus. Additionally, they can simultaneously weigh the impact of the three resources on the study area. In addition, these methods also consider the extension of the water–energy–food-related system, that is, the impact of external factors such as economy, society, environment, and related policies on it.

“Symbiosis” was originally an important basic concept in biological sciences. In 1879, it was first proposed by German biologist Anton de Bery who believed that symbiosis is the living together of different species of organisms [42]. “Symbiosis theory” is one of the basic principles of ecology, describing the nutritional connection of living organisms [43]. Scott [44], Margulis [45,46,47] and Golf [48] enriched and developed Anton de Bery’s symbiosis thought, and gradually formed a systematic symbiosis theory. With the further in-depth study of symbiosis issues, the symbiosis theory has received widespread attention in the biological community. Since the middle of the 20th century, the development of symbiosis theory has not only promoted tremendous progress in the development of biology itself, but also has gradually been widely used and developed in other fields such as industry, society, economy, and ecological environment, which has achieved remarkable results and shown its broad prospects [49,50,51,52,53,54].

However, the current academic research on the water–energy–food nexus is mostly from the perspectives of security, resilience, sustainability and synergy and is rarely from the perspective of resource symbiosis. Additionally, the symbiosis theory and system analysis method are rarely used to study the symbiosis between water, energy and food [55]. Moreover, in terms of the selection of evaluation models for the water–energy–food nexus, the static coordination degree evaluation model believes that the smaller the gap between the water, energy and food systems’ security level and the ideal value is, the more coordinated the water–energy–food nexus is. The disadvantage of the static model is that the ideal level is difficult to determine and the interaction between water, energy and food has not been considered; The dynamic coordination degree evaluation model believes that the closer the evolution speed of water, energy and food systems is, the more coordinated the water–energy–food nexus is, which ignores the security level of the existing water, energy, and food systems. In fact, water–energy–food represents a symbiotic combination. While studying the level of water, energy and food security, it is also necessary to take the interaction between the three systems into account. Therefore, the relationship between water, energy and food can be analyzed by analogy with the relationship between the population in biology, and the Lotka–Volterra model can be applied to the study of the symbiotic and coordinated development of water, energy and food.

Based on the above analysis, this article draws on ecological symbiosis theory and developmental psychological ecological systems theory to construct a conceptual model of water–energy–food ecosystem (hereinafter referred to as WEF ecosystem) from the perspective of water–energy–food symbiosis. Then, the two-step measurement method of symbiotic coordination is used to study the symbiotic relationship and the grade of symbiotic coordination of the WEF ecosystem in the Yangtze River Basin. That is, the first step is to use the Lotka–Volterra symbiotic evolution model to calculate the WEF ecosystem symbiotic index to analyze the water, energy and food symbiotic relationship and security grade, and to determine whether the WEF ecosystem symbiotic security grade of each province or municipality in the Yangtze River Basin has entered the health threshold; The second step is to measure the symbiotic level index of each province or municipality where the WEF ecosystem is healthily symbiotic, and to judge the symbiotic level of water, energy and food in the healthily symbiotic provinces or municipalities. Then, the BP neural networks–decision making trial and evaluation laboratory–game theory combination weight (hereinafter referred to as BP-DEMATEL-GTCW) key influencing factors identification model was constructed to identify the key influencing factors that affect the symbiotic security of the WEF ecosystem. Finally, based on the evaluation results, this paper analyzes key influencing factors of symbiotic security and puts forward countermeasures and suggestions, which can provide decision-making reference for the management of water, energy and food in the Yangtze River Basin.

## 2. Methods

**Figure 1:**
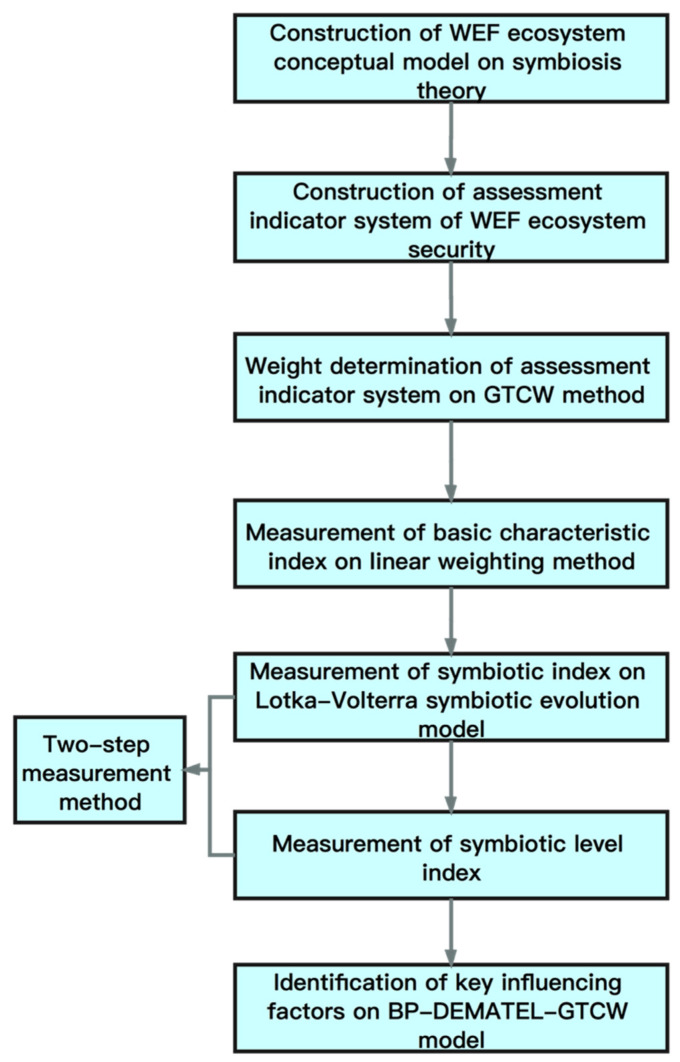
Flow chart.

According to the technical idea of this article, the research method is divided into the following 6 steps, which is shown in Figure 1. Firstly, we build a conceptual model of the WEF Ecosystem based on the perspective of symbiosis, using symbiosis theory. Secondly, we construct the assessment indicator system of WEF ecosystem security based on the conceptual model. Thirdly, we use the game theory combination weight (hereinafter referred to as GTCW) method to weight each microsystem’s indexes in the WEF ecosystem and use the linear weighting method to measure the basic characteristic index of each microsystem. Fourthly, we carry out the first stage measurement of the two-step measurement method of symbiotic coordination, that is, using the Lotka–Volterra symbiotic evolution model to measure the WEF ecosystem symbiotic index. Fifthly, we carry out the second stage measurement of the two-step measurement method of symbiotic coordination, that is, measuring the symbiotic level index of each region where the WEF ecosystem is healthily symbiotic. Sixthly, we identify the key influencing factors that affect the symbiosis safety of the WEF ecosystem by constructing the BP-DEMATEL-GTCW key influencing factor identification model. In the end, we can analyze the key influencing factors of symbiotic security based on the results of the WEF ecosystem symbiotic security evaluation and propose countermeasures to provide decision-making reference for regional water, energy and food resources management.

### 2.1. Conceptual Model of WEF Ecosystem

**Figure 2:**
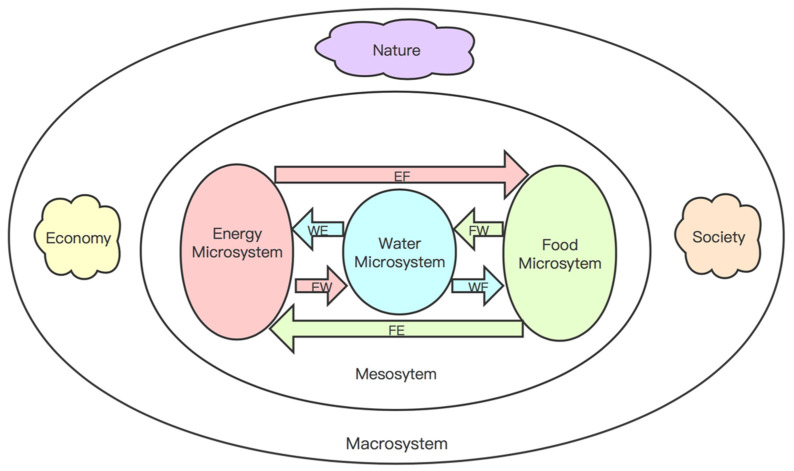
Conceptual model of WEF Ecosystem. Note: the WEF in the picture is the abbreviation of water–energy–food, WE means that water acts on energy, EW means that energy acts on water, WF means that water acts on food, FW means that food acts on water, EF means that energy acts on food, FE means that food acts on energy.

In this paper, the ecological symbiosis theory proposed by Lynn Margulis [45,46,47] and the developmental psychological ecological systems theory proposed by U Bronfenbrenner [56] are applied to the construction of the conceptual model of the WEF ecosystem and are improved according to the actual research needs of this research. The conceptual model of the WEF ecosystem based on the symbiosis theory reflects the development of each symbiotic unit that constitutes the WEF nexus nested in the symbiotic environment. Under this framework, the symbiotic relationship between water, energy, and food symbiosis units, as well as the mutual feedback relationship between the symbiotic unit and the symbiotic environment, will affect the safety of the symbiosis unit. The specific structure is shown in Figure 2.

The WEF Ecosystem consists of 3 levels: microsystem, mesosystem and macrosystem.

1. Microsystem. The microsystem in the WEF ecosystem refers to the most direct environment in which the symbiosis unit (resources such as water, energy and food) is located, and is the innermost layer of the entire WEF ecosystem. The WEF ecosystem includes three microsystems: water microsystem, energy microsystem and food microsystem. These microsystems are the basis for the symbiosis of water, energy and food. Moreover, the carrying capacity of these microsystems affects the upper limit of symbiotic security. The more positive the development of the internal quantity, quality, structure and function of the microsystem is, the greater its carrying capacity is, the smaller its vulnerability is, and the stronger the security of water–energy–food symbiosis is.
2. Mesosystem. The mesosystem in the WEF ecosystem refers to the connection and interrelationship between the water microsystem, energy microsystem, and food microsystem in the macro symbiosis environment. If there are strong and positive connections between microsystems, then the development of the entire WEF ecosystem can be optimized. On the contrary, the existence of non-positive connections between microsystems will have negative consequences.
3. Macrosystem. The macrosystem in the WEF ecosystem refers to the sum of the external symbiotic environment such as the social environment, the economic environment, and the natural environment. The macrosystem is the external driving force for the symbiosis of water, energy and food. Additionally, it affects each other with the mesosystem. The more water, energy and food microsystems can adapt to changes in the macrosystem and ensure the supply of related products and services, the greater the social, economic and environmental effects of water, energy and food resources are.

Based on the above analysis, combined with the author’s previous research [42], the operation process of the WEF ecosystem is very consistent with the principle of the WEF ecosystem Lotka–Volterra symbiotic evolution model which will be described in the next section.

### 2.2. Assessment Indicator System of WEF Ecosystem Security

**Figure 3:**
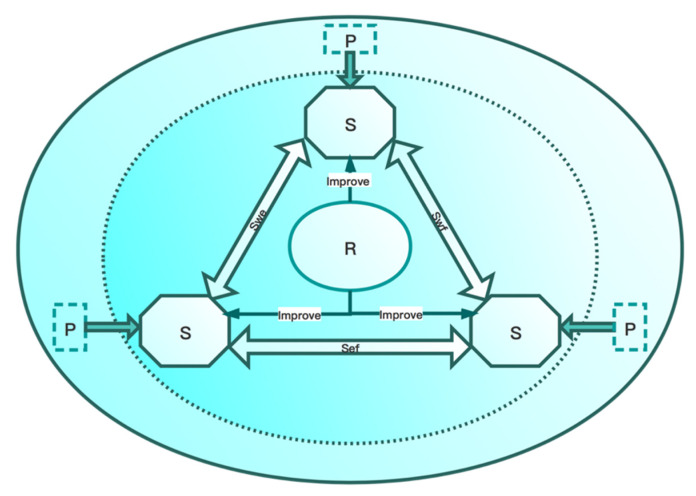
The graph of comprehensive importance degree of assessment indicators. Note: Swe, Swf, and Sef represent the interactions between water–energy, water–food, and energy–food, respectively.

Based on the conceptual model of the WEF ecosystem from the perspective of symbiosis, this paper adopts a pressure–state–response (PSR) model that can reflect the comprehensive dynamic transmission mechanism and change process generated by multiple factors on the system to study the symbiotic security of the regional WEF ecosystem. The specific framework is shown in Figure 3. Among them, pressure represents the factors that threaten the security of water, energy and food microsystems by the macrosystem, and reflects the direct cause of changes in regional water, energy and food security; State represents the security state of the 3 microsystems in the WEF ecosystem; Response represents the efforts and measures made by the human society to improve the security of the WEF system. The external macrosystem affects the state of the water, energy and food microsystems by the use of resources and the discharge of pollutants. The government and other relevant departments take corresponding measures to improve the security level of the WEF ecosystem according to the pressure on the microsystems and its own security state. In addition, the PSR model can also reflect the interaction between the various micro-systems in the WEF ecosystem.

Based on the analysis of the symbiotic security of the WEF ecosystem using the PSR model, this paper constructs an assessment indicator system of WEF ecosystem security from the 3 dimensions of pressure, state and response for each microsystem. Among them, the indicators under the pressure dimension include the pressure on the use of water, energy and food, as well as the pressure caused by the discharge of pollutants in the use of the above resources. These indicators are all negative indicators. The indicators under the state dimension mainly include indicators that reflect the regional water, energy, and food carrying capacity. Most of these indicators are positive indicators. The indicators under the response dimension include relevant indicators that reflect the countermeasures taken by the government and other relevant departments to prevent, improve and adapt to changes in the resource security state. These countermeasures include improving water resources security by afforestation and increasing water-saving irrigation rates, increasing the level of energy security by increasing investment in energy-related industries, and increasing the level of food security by building dams and increasing investment in agricultural machinery. In addition, these indicators are all positive indicators. The specific assessment indicator system of WEF ecosystem security is shown in Table 1.

### 2.3. Measurement of the Basic Characteristic Index Based on GTCW Model

This paper selects the entropy method and the CRITIC method to calculate the weights of the indicator system of the 3 basic characteristic indexes. Then, this paper uses the standardized values of related indicators and the weight of each indicator in the assessment indicator system of WEF ecosystem security to calculate the final comprehensive evaluation value of the 3 basic characteristic indexes.

#### 2.3.1. Dimensionless Standardization of Indicator Values

In order to eliminate the influence of the dimension and its unit so as to make each indicator can be converted into a value that can be directly added or subtracted, it is necessary to carry out a dimensionless standardization process on the original data. There are 11 negative indicators in the assessment indicator system of WEF ecosystem security established in this paper, and the rest are positive indicators. The larger the positive indicator value is or the smaller the negative indicator value is, the larger the corresponding indicator value is. The non-dimensional standardization processing formula of the positive index is as follows:

(1) $$X_{ij}=\frac{x_{ij}-minx_{ij}}{maxx_{ij}-minx_{ij}}$$

The non-dimensional standardization processing formula of the negative index is as follows:

(2) $$X_{ij}=\frac{maxx_{ij}-x_{ij}}{maxx_{ij}-minx_{ij}}$$

In the formula, $x_{ij}$ represents the original value of the *j*-th indicator in the *i*-th region; $maxx_{ij}$ represents the maximum value of the sample value under the *j*-th index; $minx_{ij}$ represents the minimum value of the sample value under the *j*-th index; $X_{ij}$ is the dimensionless standardized value of the *j*-th indicator in the *i*-th region.

#### 2.3.2. Weight Determination Based on the Entropy Weight Method

The entropy weight method is a mathematical method to determine the objective weight of indicators according to the degree of indicator data’s dispersion.

The smaller the information entropy of the evaluation index is, the greater the degree of the indicator value’s variation is, the more information it can provide, the greater the impact on the comprehensive evaluation is, and the greater the weight of the indicator is. The steps of entropy weight method to calculate indicator’s weight are as follows:

**Step 1**: According to the standardized decision matrix, we calculate the information entropy of the *j*-th index $E_{j}$:

(3) $$E_{j}=-\frac{1}{\ln\left(n\right)}\overset{j}{\underset{i=1}{\sum }}(f_{ij}lnf_{ij})$$

In the formula, $f_{ij}=X_{ij}/\sum_{i=1}^{j}X_{ij}$; We set that $f_{ij}lnf_{ij}=0$ when $f_{ij}=0$.

**Step 2**: We calculate the entropy weight of each indicator $w1_{j}$:

(4) $$w1_{j}=\frac{1-E_{j}}{\sum_{j=1}^{n}\left(1-E_{j}\right)}=\frac{D_{j}}{\sum_{j=1}^{n}\left(D_{j}\right)}$$

In the formula, $D_{j}$ is the indicator difference degree; $D_{j}=1-E_{j}$.

**Step 3**: We determine the weight of objective indicators $W1_{k}$:

(5) $$W1_{k}=\left(w1_{1},w1_{2},\ldots ,w1_{n}\right)$$

#### 2.3.3. Weight Determination Based on the CRITIC Method

The criteria importance though intercriteria correlation (hereinafter referred to as CRITIC) method is a mathematical method to comprehensively determine the objective weight of indicators based on the conflict between contrast intensity and evaluation indicators. The contrast intensity represents the size of the difference in the value of the evaluation schemes of the same indicator, and its manifestation is the standard deviation. The larger the standard deviation is, the greater the value difference of the schemes within the same indicator is. The conflict between the evaluation indicators is based on the correlation between the indicators. The stronger the positive correlation between the indicators is, the lower the conflict between the indicators is. The steps of the CRITIC method to calculate indicator’s weight are as follows:

**Step 1**: We quantitatively measure the contrast intensity of evaluation indicators. The formula of the standard deviation $S_{j}$ of the *j*-th indicator is as follows:

(6) $$\{\begin{array}{c} {\bar{X}}_{j}=\frac{1}{n}\overset{n}{\underset{i=1}{\sum }}X_{ij}\\ S_{j}=\sqrt{\frac{\sum_{i=1}^{n}(X_{ij}-{\bar{X}}_{j})}{n-1}} \end{array}$$

**Step 2**: We quantitatively measure the conflict between evaluation indicators. The formula of the correlation coefficient is as follows:

(7) $$R_{j}=\overset{n}{\underset{i=1}{\sum }}(1-r_{ij})$$

In the formula, $r_{ij}$ indicates the correlation coefficient between the evaluation indicator *i* and *j*.

**Step 3**: We quantitatively measure the amount of information of evaluation indicators. The greater the amount of information $C_{j}$ contained in the *j*-th evaluation indicator is, the greater the relative importance of the indicator is. The formula is as follows:

(8) $$C_{i}=S_{j}*R_{j}$$

**Step 4**: We calculate the weight of the *j*-th indicator $w2_{j}$:

(9) $$w2_{j}=\frac{C_{j}}{\sum_{j=1}^{m}C_{i}}$$

**Step 5**: We determine the weight of objective indicators $W2_{k}$:

(10) $$W2_{k}=\left(w2_{1},w2_{2},\ldots ,w2_{j}\right)$$

#### 2.3.4. Combination Weight Determination Based on the GTCW Method

Since the entropy weight method and the CRITIC method have different weight determination principles, using them separately to determine the weight will have certain limitations in the final result. Specifically, the entropy weight method does not consider the correlation, conflict and contrast strength between indicator information, while the CRITIC method does not consider the degree of indicator data’s dispersion. These two weighting methods can make up for each other’s deficiencies. Therefore, this paper uses the game theory method to integrate the entropy weigh method and the CRITIC method, so that the weight of the indicator can be comprehensively determined. Furthermore, the relevance, dispersion and relative intensity of the indicator data information can be fully considered. Combining multiple objective weighting methods by game theory can make the final weighting result tend to a more balanced state and ensure the scientific rationality of the indicator weight. The steps of the GTCW method to calculate indicator’s weight are as follows:

**Step 1**: Entropy method and CRITIC method are used to weight the indicators respectively, and a basic weight vector set $wk_{j}=\left\{wk_{1},wk_{2},\ldots ,wk_{j}\right\},\;k=1,2$, is constructed. Any linear combination between the above 2 different vectors is:

(11) $$w=\overset{2}{\underset{k=1}{\sum }}\alpha_{k}w_{k}^{T}\;(\alpha_{k}>0,\overset{2}{\underset{k=1}{\sum }}\alpha_{k}=1)$$

In the formula, *w* is a possible weight vector in the basic weight vector set; $\alpha_{k}$ is the linear combination coefficient.

**Step 2**: We use game theory to optimize the two linear combination coefficients $\alpha_{k}$, so that the deviation between w and each $w_{km}$ is the smallest, namely

(12) $$min{∥\overset{2}{\underset{j=1}{\sum }}\alpha_{j}w_{j}^{T}-w_{i}∥}_{2}\left(i=1,2\right)$$

The optimal first derivative condition of the above formula can be converted to the following formula:

(13) $$\left[\begin{array}{cc} w_{1}w_{1}^{T} & w_{1}w_{2}^{T}\\ w_{2}w_{1}^{T} & w_{2}w_{2}^{T} \end{array}\right]\left[\begin{array}{c} \alpha_{1}\\ \alpha_{2} \end{array}\right]=\left[\begin{array}{c} w_{1}w_{1}^{T}\\ w_{2}w_{2}^{T} \end{array}\right]$$

**Step 3**: According to the above process, $\left(\alpha_{1},\alpha_{2}\right)$ is obtained and then is normalized, that is, the game theory combination weight $w_{j}$ of the *j*-th index is calculated:

(14) $$w_{j}=\frac{\left|\alpha_{k}\right|}{\sum_{k=1}^{2}\left|\alpha_{k}\right|}$$

**Step 4**: We calculate the game theory combination weight of objective indicators $W_{k}$:

(15) $$W_{k}=\left(w_{1},w_{2},\ldots ,w_{j}\right)$$

#### 2.3.5. Calculation of Basic Characteristic Index

We use the standardized values of related indicators and the weights of each indicator in the assessment indicator system of WEF ecosystem security to calculate the final comprehensive evaluation value of the 3 basic characteristic indexes. The calculation formula is as follows:

(16) $$Z_{i}=\overset{n}{\underset{j=1}{\sum }}\left(w_{j}X_{ij}\right)$$

In the formula, *Z* represents each province’s or municipality’s security level index of the water microsystem *W*, security level index of the energy microsystem *E*, and security level index of the food microsystem *F*; $w_{j}$ is the weight of each indicator; $X_{ij}\;$is the value of the *j*-th index of the *i*-th region after non-dimensional standardization.

### 2.4. Two-Step Measurement Method of Symbiotic Coordination

After using the GTCW method to measure the basic characteristic index of the 3 microsystems in the WEF ecosystem, we use the two-step measurement method of symbiotic coordination to study the symbiotic relationship and the grade of symbiotic coordination of the WEF ecosystem. That is, the first step is to use the Lotka–Volterra symbiotic evolution model to calculate the WEF ecosystem symbiotic index to analyze the water, energy and food symbiotic relationship and security grade, and to determine whether the WEF ecosystem symbiotic security grade of each region has entered the health threshold; The second step is to measure the symbiotic level index of each region where the WEF ecosystem is healthily symbiotic, and to judge the symbiotic level of water, energy and food in the healthily symbiotic region.

#### 2.4.1. Measurement of Symbiotic Index

We assume that W(t),E(t),F(t) respectively represent the security level of water, energy and food microsystem, $\gamma_{i}\left(>0,i=W,E,F\right)$ represents the net growth rate of the stability level of microsystem *i*, and $K_{i}\left(i=W,E,F\right)$ represents the highest security and stability level of microsystem *i*. Then, from the perspective of symbiosis, the evolutionary dynamics equations of the water, energy, and food microsystem in the WEF ecosystem, namely the Lotka–Volterra symbiotic evolution model are as follows:

(17) $$\left\{ \begin{array}{c} \frac{dW\left(t\right)}{dt}=F_{W}\left(W,E,F\right)=\gamma_{W}W\left(t\right)\left(1-\frac{W\left(t\right)}{K_{W}}\right)+\gamma_{W}\theta_{WE}\frac{W\left(t\right)E\left(t\right)}{K_{E}}+\gamma_{W}\theta_{WF}\frac{W\left(t\right)F\left(t\right)}{K_{F}}\\ \frac{dE\left(t\right)}{dt}=F_{E}\left(W,E,F\right)=\gamma_{E}E\left(t\right)\left(1-\frac{E\left(t\right)}{K_{E}}\right)+\gamma_{E}\theta_{EW}\frac{E\left(t\right)W\left(t\right)}{K_{W}}+\gamma_{E}\theta_{EF}\frac{E\left(t\right)F\left(t\right)}{K_{F}}\\ \frac{dF\left(t\right)}{dt}=F_{F}\left(W,E,F\right)=\gamma_{F}F\left(t\right)\left(1-\frac{F\left(t\right)}{K_{F}}\right)+\gamma_{F}\theta_{FW}\frac{F\left(t\right)W\left(t\right)}{K_{W}}+\gamma_{F}\theta_{FE}\frac{F\left(t\right)E\left(t\right)}{K_{E}} \end{array}\right.$$

In the formula, $\theta_{WE},\theta_{WF}$ are the coefficient reflecting the effect of energy and food microsystem on water micro-system respectively; $\theta_{EW},\theta_{EF}$ are the coefficient reflecting the effect of water and food microsystem on energy microsystem respectively; $\theta_{FW},\theta_{FE}$ are the coefficient reflecting the effect of water and energy microsystem on the food microsystem respectively; The size of these coefficients indicates the size of the symbiotic effect. Specifically, $\theta_{ij}>0\left(i=W,E,F;j=W,E,F\right)$ indicates the promotion effect, and $\theta_{ij}<0$ indicates the inhibition effect; $\gamma_{W}W\left(t\right),\gamma_{E}E\left(t\right),\gamma_{F}F\left(t\right)$ respectively represent the development trend of water, energy and food microsystem; $1-\frac{W\left(t\right)}{K_{W}},1-\frac{E\left(t\right)}{K_{E}},1-\frac{F\left(t\right)}{K_{F}}$ are Logistic coefficients, which represent the retarding effect on the growth of its own scale caused by the water, energy and food microsystem’s consumption of limited resources. When the water, energy and food microsystem interact, the growth rate of each microsystem in the WEF ecosystem is not only affected by its own scale, but also related to the scale of other microsystems in the WEF ecosystem. Therefore, the development of water, energy, and food microsystem will be affected by the symbiotic competition coefficient.

We can obtain these coefficients between the various microsystems in the WEF ecosystem by the parameter estimation method, and then obtain the symbiotic index between the microsystems. The calculation formula for the symbiotic index of water, energy and food microsystem is as follows:

(18) $$S\left(k\right)=\frac{\theta_{WE}\left(k\right)+\theta_{EW}\left(k\right)+\theta_{WF}\left(k\right)+\theta_{FW}\left(k\right)+\theta_{EF}\left(k\right)+\theta_{FE}\left(k\right)}{\sqrt{\theta_{WE}^{2}\left(k\right)+\theta_{EW}^{2}\left(k\right)+\theta_{WF}^{2}\left(k\right)+\theta_{FW}^{2}\left(k\right)+\theta_{EF}^{2}\left(k\right)+\theta_{FE}^{2}\left(k\right)}}$$

According to the nature of the symbiosis function, we can find that $S_{ijk}\in \left[-\sqrt{6},\sqrt{6}\right]$, and different value ranges of $S_{ijk}$ have different meanings. The specific contents are shown in Table 2.

When $S_{ijk}\in \left[-1,1\right]$, there are two situations: first, there is a certain degree of complementarity between the 3 microsystems, each microsystem may transform to the side that is beneficial to it; Secondly, one or two of the microsystems have too strong inhibitory effects to turn them in a direction that is harmful to themselves.

#### 2.4.2. Measurement of Symbiotic Level Index

For the study on the symbiotic security of the WEF ecosystem, it is not enough to measure the symbiotic index in the first step of the two-step measurement of symbiotic coordination. The reason is that for the regions whose WEF ecosystems’ symbiotic security grade has reached a healthy grade, although they have reached a healthy stage in terms of “qualification”, their development levels are not consistent. Some are still in the initial stage of development, and their symbiotic stability is not high enough; some are already in the mature stage, and their symbiotic stability is relatively high. In order to further measure the level of symbiotic development of the areas where the WEF ecosystem’s symbiotic security grade has reached a health grade, it is necessary to comprehensively measure factors such as the security level of water–energy–food, the degree of water–energy–food symbiosis, and the level of balanced development of water–energy–food. Therefore, the second step of the two-step measurement of symbiotic coordination is also needed, that is, the measurement of the symbiotic level index. We construct the WEF ecosystem symbiotic level index $G_{i}\left(k\right)$, and its calculation formula is as follows:

(19) $$G_{i}\left(k\right)=\sigma_{i}\left(k\right)\tau_{i}\left(k\right){\tilde{G}}_{i}\left(k\right)$$

In the formula, $G_{i}\left(k\right)\;$represents the symbiotic level index of the regional WEF ecosystem, which reflects the level of stability of the WEF ecosystem’s coordinated development.

Among them, the calculation formula of the symbiotic coefficient $\sigma_{i}\left(k\right)$ is as follows:

(20) $$\sigma_{i}\left(k\right)=\frac{\sqrt{2}+S_{i}\left(k\right)}{2\sqrt{2}}$$

In the formula, $\sigma_{i}\left(k\right)$ reflects the degree of symbiosis between the 3 microsystems of the regional WEF ecosystem. Because $S_{i}\left(k\right)\in \left[-\sqrt{2},\sqrt{2}\right]$, $\sigma_{i}\left(k\right)\in \left[0,1\right]$. Moreover, $\sigma_{i}\left(k\right)$ is the standardized processing for the symbiotic index $S_{i}\left(k\right)$.

Furthermore, the calculation formula of the equilibrium coefficient $\tau_{i}\left(k\right)$ is as follows:

(21) $$\tau_{i}\left(k\right)=1-\frac{\left|W_{i}\left(k\right)-E_{i}\left(k\right)\right|+\left|W_{i}\left(k\right)-F_{i}\left(k\right)\right|+\left|E_{i}\left(k\right)-F_{i}\left(k\right)\right|}{W_{i}\left(k\right)+E_{i}\left(k\right)+F_{i}\left(k\right)}$$

In the formula, $\tau_{i}\left(k\right)$ represents the equilibrium coefficient between the 3 microsystems of the regional WEF ecosystem, that is, the degree of coordination achieved by the interaction of water, energy and food. Moreover,$\;\tau_{i}\left(k\right)\in \left(0,1\right]$. The larger the equilibrium coefficient is, the better the balance between the 3 microsystems is, and the smaller the difference is. When $W_{i}\left(k\right)=E_{i}\left(k\right)=F_{i}\left(k\right)$, $\tau_{i}\left(k\right)=1$. Refer to existing research and divide the equilibrium coefficient into several levels to judge the equilibrium grade of the WEF ecosystem. See Table 3 for details.

Moreover, the calculation formula for the comparable total ${\tilde{G}}_{i}\left(k\right)$ of the 3 microsystems of the regional WEF ecosystem is as follows:

(22) $${\tilde{G}}_{i}\left(k\right)=\frac{\frac{W_{i}\left(k\right)+E_{i}\left(k\right)+F_{i}\left(k\right)}{a_{i}\left(k\right)}}{\frac{1}{nm}\sum_{i=1}^{n}\sum_{k=1}^{m}\frac{W_{i}\left(k\right)+E_{i}\left(k\right)+F_{i}\left(k\right)}{a_{i}\left(k\right)}}$$

In the formula, when *m* and *n* are both 1, ${\tilde{G}}_{i}\left(k\right)=1$; ${\tilde{G}}_{i}\left(k\right)\in \left(0,\;nm\right]$; *i* represents each region, i=1,2,……,n; *k* represents the year, k=1,2,……,m; $a_{i}\left(k\right)$ represents the GDP of region *i* in the *k*-th year, and this value is normalized.

### 2.5. BP-DEMATEL-GTCW Key Influencing Factor Identification Model

The decision-making trial and evaluation laboratory (hereinafter referred to as DEMATEL) is a modeling method proposed by an American scholar in 1971 to analyze the relationship between various factors in the system. This method uses the combination of graph theory and matrix tools and establishes a direct correlation matrix based on the logical relationship between the factors in the system, in order to calculate the effect degree, affected degree, prominence degree and cause degree of each factor. Based on this, the type of each factor can be derived, so as to quantify the degree of mutual influence between the factors and the importance of each factor in the system [57,58]. This paper chooses to use BP neural network method to improve the traditional DEMATEL model and obtains the correlation matrix through the calculation of weights, thereby increasing the credibility of the key influencing factor identification results and analysis. Then, we calculate the prominence degree and cause degree of each input factor according to the traditional DEMATEL method and use the GTCW method and linear weighting method to calculate the comprehensive importance degree. The specific steps of the BP-DEMATEL-GTCW model to identify key influencing factors are as follows:

**Step 1**: We establish the matrix *x* and *y* of influencing factors and affected factors. In detail, we let the influencing factor matrix be $x={\left(x_{ij}\right)}_{m\times n}$ and let the affected factor matrix be $y={\left(y_{ik}\right)}_{m\times t}$. Then, we obtain *X* and *Y* after normalizing them. Among them, *m* is the sample number of the model. Additionally, *n* and *t* respectively represent the number of influencing factors and affected factors. i=1,2,…,m,j=1,2,…,n,k=1,2,…,t.

**Step 2**: We calculate the weight matrix $W_{t}$. In detail, we let *Y* be the output vector and *X* be the input vector and establish a BP neural network by MATLAB 2018a, in order to obtain the weight matrix ${\left(W_{t}\right)}_{n\times l}$ of the input layer and the hidden layer, and the weight matrix ${\left(w_{t}\right)}_{l\times k}$ of the hidden layer and the output layer. Among them, *l* is the number of neuron nodes in the hidden layer.

**Step 3**: We calculate the overall weight vector ω. In detail, we take the absolute value of the two weight matrixes ${\left(W_{t}\right)}_{n\times l}$ and ${\left(w_{t}\right)}_{l\times k}$ obtained in step 2. Then, we calculate the overall weight vector $\omega ={\left|W\right|}^{*}\left|w\right|$, where $\omega =\omega_{n\times k}$. After calculating and transposing, the overall weight vector $\omega =\left(w_{1},w_{2},\ldots ,w_{n}\right)$ is obtained.

**Step 4**: We calculate the direct correlation matrix B between the influencing factors:

(23) $$B={\left(b_{ij}\right)}_{n\times n}=\left(\begin{array}{cc} \begin{array}{cc} b_{11} & b_{12}\\ b_{21} & b_{22} \end{array} & \begin{array}{cc} \cdots & b_{1n}\\ \cdots & b_{2n} \end{array}\\ \begin{array}{cc} \vdots & \vdots \\ b_{n1} & b_{n2} \end{array} & \begin{array}{cc} \ddots & \vdots \\ \cdots & b_{nn} \end{array} \end{array}\right)$$

In the formula, $b_{ii}=0$,$b_{ij}=\frac{w_{i}}{w_{j}}$ indicates the importance degree of the *i*-th influencing factor relative to the *j*-th influencing factor. Moreover, when $w_{j}=0$, $b_{ij}=0$.

**Step 5**: According to the principle of the sum of all values, we normalize the direct incidence matrix *X*:

(24) $$X={\left(x_{ij}\right)}_{n\times n}=\frac{1}{max\sum_{1\leq i\leq n,j-1}^{n}b_{ij}}\cdot B$$

**Step 6**: We calculate the full incidence matrix T to grasp the direct and indirect influence factors between factors:

(25) $$T=X+X^{2}+\ldots +X^{n}=X{\left(I-X\right)}^{-1}$$

In the formula, ${\left(I-X\right)}^{-1}$ is the inverse of I−X, and *I* is the unit matrix.

**Step 7**: We establish a causality diagram, define *D* as the sum of the rows of *T*, and define *R* as the sum of the columns of *T*:

(26) $$T_{i}={\left(t_{ij}\right)}_{n\times n}$$

(27) $$D_{i}={\left(t_{i\cdot }\right)}_{n\times 1}={\left(\overset{n}{\underset{j=1}{\sum }}t_{ij}\right)}_{n\times 1}$$

(28) $$R_{i}={\left(t_{\cdot j}\right)}_{1\times n}={\left(\overset{n}{\underset{i=1}{\sum }}t_{ij}\right)}_{1\times n}$$

In the formula,$\;D_{i}$ refers to the comprehensive influence value of other influencing factors on influencing factor *i*, which is called the effect degree; $R_{i}$ refers to the comprehensive influence value of influencing factor *i* on other influencing factors, which is called affected degree; We let $P_{i}=R_{i}+D_{i}$ be the prominence degree of indicator *i*. The value reflects the importance of the indicator. The larger the value is, the stronger the importance of the indicator is; We let $Q_{i}=R_{i}-D_{i}$_i be the cause degree of indicator *i*. The value reflects the relevance degree of the indicator. The larger the value is, the stronger the relevance of the indicator is. According to its value, it can be divided into cause group or result group. If the value of indicator *i*’s$\;D_{i}-R_{i}$ is greater than 0, the indicator will be divided into reason groups. If the value of indicator *i*’s$\;D_{i}-R_{i}$ is less than 0, the indicator will be divided into the result group. Among all the influencing factors, the factors in the result group are reflected as the influence results of the factors in the cause group.

**Step 8**: We use the GTCW method to give weight to prominence degree and cause degree to calculate the comprehensive importance degree $\rho_{i}$:

(29) $$\rho_{i}=E_{z}P_{i}+E_{y}Q_{i},\left(i=1,2,\ldots ,n\right)$$

In the formula, $E_{z}$ and $E_{y}$ are the weights of the prominence degree $P_{i}$ and cause degree $Q_{i}$ of each influencing factor respectively.

**Step 9**: We determine the key influencing factors of the symbiotic coordination of the WEF ecosystem. In detail, we find out all influencing factors whose comprehensive importance degree is greater than 0, and sort them according to their numerical value. The top influencing factors in comprehensive importance play key roles, and they will be determined as key influencing factors.

## 3. Results and Discussion

### 3.1. Study Area and Data Resource

The Yangtze River is the mother river of the Chinese nation, whose length is about 6300 km. The vast area through which the mainstream and tributaries of the Yangtze River flow is the Yangtze River Basin. Moreover, it spans the three major economic regions of eastern, central and western China, with a basin area of about 1.8 million km^2^, accounting for 18.8% of China’s land area [59]. In terms of water resources, the Yangtze River Basin is the most water-rich basin in China, with total water resources of 975.5 billion m^3^, accounting for about 36% of the total river runoff in China. However, there is still water shortage in some areas in the Yangtze River Basin. In terms of energy, the Yangtze River itself is a huge treasury of hydropower resources. The clean and low-carbon development of energy in the Yangtze River Basin is generally better than the national average; The energy consumption per unit of GDP in the Yangtze River Basin is 12%, which is lower than the national average, and indicates that the energy efficiency level is higher [60]. However, the Yangtze River Basin also has energy security issues that restrict development. In terms of food, the Yangtze River Basin is an important grain production base in China, with a food output of 1.63 tons, accounting for 32.5% of the national food output [61]. However, the situation of food supply and demand in the Yangtze River Basin is severe.

**Figure 4:**
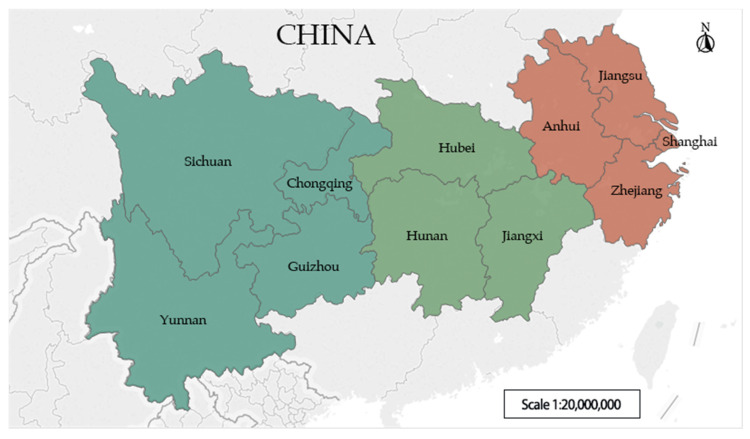
Study area.

In the selection of research scope in the Yangtze River Basin, some studies specifically select a single typical city in the Yangtze River Basin [62], and some studies select the sub-basins below the Yangtze River Basin [63]. From the perspective of water–energy–food symbiosis, this research studies the degree of symbiotic coordination of the WEF ecosystem in the Yangtze River Basin, and takes into account the social, economic and ecological environment. Therefore, in the definition of the study area, taking the river basin as the foundation and the Yangtze River as the link, we select 11 provinces or municipalities as the research objects, including Shanghai, Jiangsu, Zhejiang and Anhui in the upper reaches of the Yangtze River, Jiangxi, Hubei and Hunan in the middle reaches of the Yangtze River, and Chongqing, Sichuan, Guizhou and Yunnan in the lower reaches of the Yangtze River. The specific research area is shown in Figure 4.

Most of the data in this study come from the “China Statistical Yearbook” (2009–2018) and the provincial statistical yearbooks. They also refer to the “China Environmental Statistical Yearbook”, “China Soil and Water Conservation Bulletin”, “China Energy Statistical Yearbook” (2009–2018) and the provincial water resources bulletins and environmental status bulletins.

### 3.2. Results of Symbiotic Index

In order to calculate the symbiotic coefficients of the three main bodies in the Lotka–Volterra symbiotic evolution model, we use the grey estimation method to write Equation (17) in a general form:

(30) $$\{\begin{array}{c} \frac{dW}{dt}=F_{1}\left(W,E,F\right)=W\left(a_{0}+a_{1}W+a_{2}E+a_{3}F\right)\\ \frac{dE}{dt}=F_{2}\left(W,E,F\right)=Y\left(b_{0}+b_{1}W+b_{2}E+b_{3}F\right)\\ \frac{dF}{dt}=F_{3}\left(W,E,F\right)=Z\left(c_{0}+c_{1}W+c_{2}E+c_{3}F\right) \end{array}$$

Taking the water microsystem as an example, we set $W^{\left(0\right)}=\left\{w^{\left(0\right)}\left(i\right),i=1,2,\ldots ,n\right\}$ as a non-negative original sequence and build a sequence into a model compatible with differential, difference and approximate exponential laws, which is called the gray model. Assuming that the time interval of the original sequence is small enough, we take the unit time interval, that is,$\;\frac{dW}{dt}=W\left(t+1\right)-W\left(t\right)$. From the mapping relationship between the gray derivative and even logarithm in gray theory, we can take the background value at time *t* $\frac{W_{\left(t+1\right)}+W_{\left(t\right)}}{2},\frac{E_{\left(t+1\right)}+E_{\left(t\right)}}{2},\frac{F_{\left(t+1\right)}+F_{\left(t\right)}}{2}$, then Equation (30) is:

(31) $$\{\begin{array}{c} W\left(t+1\right)-W\left(t\right)=a_{0}\frac{W_{\left(t+1\right)}+W_{\left(t\right)}}{2}+a_{1}{\left[\frac{W_{\left(t+1\right)}+W_{\left(t\right)}}{2}\right]}^{2}\\ +a_{2}\frac{W_{\left(t+1\right)}+W_{\left(t\right)}}{2}\frac{E_{\left(t+1\right)}+E_{\left(t\right)}}{2}+a_{3}\frac{W_{\left(t+1\right)}+W_{\left(t\right)}}{2}\frac{F_{\left(t+1\right)}+F_{\left(t\right)}}{2}\\ E\left(t+1\right)-E\left(t\right)=b_{0}\frac{E_{\left(t+1\right)}+E_{\left(t\right)}}{2}+b_{1}\frac{W_{\left(t+1\right)}+W_{\left(t\right)}}{2}\frac{E_{\left(t+1\right)}+E_{\left(t\right)}}{2}\\ +b_{2}{\left[\frac{W_{\left(t+1\right)}+W_{\left(t\right)}}{2}\right]}^{2}+b_{3}\frac{E_{\left(t+1\right)}+E_{\left(t\right)}}{2}\frac{F_{\left(t+1\right)}+F_{\left(t\right)}}{2}\\ F\left(t+1\right)-F\left(t\right)=c_{0}\frac{F_{\left(t+1\right)}+F_{\left(t\right)}}{2}+c_{1}\frac{W_{\left(t+1\right)}+W_{\left(t\right)}}{2}\frac{F_{\left(t+1\right)}+F_{\left(t\right)}}{2}\\ +c_{2}\frac{E_{\left(t+1\right)}+E_{\left(t\right)}}{2}\frac{F_{\left(t+1\right)}+F_{\left(t\right)}}{2}+c_{3}{\left[\frac{F_{\left(t+1\right)}+F_{\left(t\right)}}{2}\right]}^{2} \end{array}$$

Then, we substitute t=1,2,…,n−1 into Equation (31) to obtain three equations:

(32) $$\{\begin{array}{c} Y_{1n}=X_{1}\hat{a}\\ Y_{2n}=X_{2}\hat{b}\\ Y_{3n}=X_{3}\hat{c} \end{array}$$

According to the least squares criterion, the four parameters involved in the discrete equation system can be obtained:

(33) $$\{\begin{array}{c} \hat{a}={\left[a_{0},a_{1},a_{2},a_{3}\right]}^{T}={\left(X_{1}^{T}X_{1}\right)}^{-1}X_{1}^{T}Y_{1n}\\ \hat{b}={\left[b_{0},b_{1},b_{2},b_{3}\right]}^{T}={\left(X_{2}^{T}X_{2}\right)}^{-1}X_{2}^{T}Y_{2n}\\ \hat{c}={\left[c_{0},c_{1},c_{2},c_{3}\right]}^{T}={\left(X_{3}^{T}X_{3}\right)}^{-1}X_{3}^{T}Y_{3n} \end{array}$$

Comparing Equation (17) with Equation (31), we can obtain $\gamma_{W}=a_{0},K_{W}=-\frac{a_{0}}{a_{1}},\theta_{WE}=-\frac{a_{2}}{a_{1}},\theta_{WF}=-\frac{a_{3}}{a_{1}}$. In the same way, we can obtain $\gamma_{E}=b_{0},K_{E}=-\frac{b_{0}}{b_{2}},\theta_{EW}=-\frac{b_{2}}{b_{1}},\theta_{EF}=-\frac{b_{3}}{b_{2}}$ and $\gamma_{F}=c_{0},K_{F}=-\frac{c_{0}}{c_{3}},\theta_{FW}=-\frac{c_{1}}{c_{3}},\theta_{FE}=-\frac{c_{2}}{c_{3}}$. The symbiotic degree of the WEF ecosystem is calculated according to the symbiotic coefficients obtained by the above process. Then, according to Table 2, the WEF ecosystem’s symbiotic degree of 11 provinces or municipalities in the Yangtze River Basin is classified. The specific results are shown in Table 4.

From the results of the WEF ecosystem’s symbiotic index, it can be found that among the four provinces or municipalities in the lower reaches of the Yangtze River basin, Anhui’s WEF ecosystem symbiotic security is relatively poor, and it has not reached the health grade since 2008. The symbiosis of the WEF ecosystem in Jiangsu Province is the best, and it has been in a healthy grade for most of the 9 years. The state in Zhejiang is second, and the symbiotic index is higher than 1 in many years. The symbiosis state of the three provinces in the middle reaches of the Yangtze River Basin is generally not good. Among them, Hubei and Hunan have not experienced a healthy symbiosis of water, energy and food since 2010. Among the four provinces or municipalities in the middle reaches of the Yangtze River basin, the symbiotic index of the WEF ecosystem in Guizhou is better than that of the other three provinces or municipalities, which has always been positive during 2009 and 2016, but there is a downward trend. The security of WEF symbiosis in Chongqing is second. The symbiotic index of WEF ecosystems in Sichuan and Yunnan provinces is relatively close to −1 recently, and there is still a certain distance from Chongqing and Guizhou provinces. Yunnan Province has been in danger even from 2013 to 2016.

**Figure 5:**
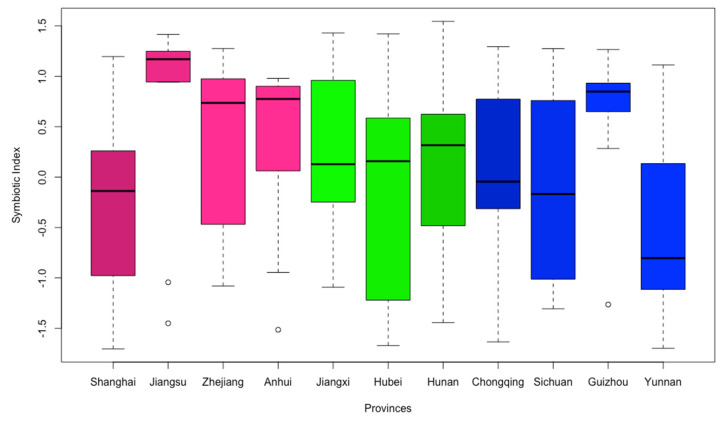
Symbiotic index of WEF ecosystem in the Yangtze River Basin.

Then, we use a box plot (Figure 5) to show the symbiosis of the WEF ecosystem in the 11 provinces or municipalities in the Yangtze River Basin, because the box plot can not only highlight the annual average symbiotic degree of each system, but also show their dispersion in a certain period of time. It can be seen from the box plot that the average symbiotic index of the WEF ecosystem in the 11 provinces or municipalities over the years is 0.1148, reaching the risk grade. This shows that the overall symbiotic security of the WEF ecosystem in the Yangtze River Basin still has a large amount of room for improvement, and the coordination of the symbiotic development of the WEF ecosystem needs to be further optimized. The length of the “box” also reflects the degree of variation in the symbiotic security of the WEF ecosystem in each region. The two provinces of Hubei and Sichuan have longer “boxes”, showing that early WEF ecosystems of these provinces have high symbiotic security and good symbiotic synergy. However, with the development of society and economy, the symbiotic index of WEF has decreased recently, and the symbiotic security grade has been fluctuating back and forth between danger and risk. The symbiotic index of Guizhou and Shanghai fluctuated greatly during the 9 years. Unlike the three provinces with longer “boxes” mentioned above, the symbiotic security of WEF ecosystems in Guizhou and Shanghai was not very satisfactory in the early period. With the effective advancement of water, energy and food resource management, symbiotic security has improved recently.

### 3.3. Results of Symbiotic Level Index

For the study of the WEF ecosystem’s symbiotic security, it is not enough to measure the symbiotic index. In order to further study the level of comprehensive symbiotic coordination in the region whose WEF ecosystem symbiotic security grade has reached a health grade, it is necessary to use Equation (22) to calculate the symbiotic level index of the regions whose WEF ecosystem symbiotic security grade has entered the health threshold on the basis of the study of the symbiotic grade. The results of the WEF ecosystem symbiotic level index are shown in Table 5. The table also includes the WEF ecosystem’s equilibrium coefficient and its grade.

Then, we analyze the symbiotic level of WEF ecosystems in the lower, middle and upper reaches of the Yangtze River. In the lower reaches of the Yangtze River, the WEF ecosystems in Shanghai did not reach a health grade until 2016. The equilibrium level of the WEF ecosystems is primarily balanced development, and the optimal management effects of water, energy and food resources are beginning to appear. Zhejiang Province reached a health grade only in 2009 and 2010, and the symbiotic level indexes in these 2 years were low, both below 11. The symbiosis of the WEF ecosystem in Jiangsu Province has been in a healthy grade for most of the 9 years, and the equilibrium level and symbiotic level show a good upward trend. In the middle reaches of the Yangtze River, from 2012 to 2013, the balance of water–energy–food symbiosis development in Jiangxi Province was relatively good, stabilizing at the primarily and intermediately development levels. In the upper reaches of the Yangtze River, the level of symbiosis in the WEF ecosystem of the provinces or municipalities in the upper reaches of the Yangtze River is as follows: Similar to the coastal areas of Shanghai, Chongqing has only entered a health grade since 2016. Its level of symbiotic coordination is higher than Shanghai. Guizhou entered a healthy state in 2012 and 2014, and the symbiotic levels both exceeded 50, which is at the top of the Yangtze River Basin. In general, for provinces and municipalities where the WEF ecosystem has entered a healthy state, it is necessary to continue to optimize the collaborative management of water, energy, and food resources to further improve the coordinated development of the regional WEF ecosystem. For provinces or municipalities that have not yet entered the health grade, it is necessary to further study and find out the key influencing factors of the decline in the symbiotic coordination of their WEF ecosystem, so that the management of water–energy–food resources can be improved in a targeted manner.

### 3.4. Identification of Key Influencing Factors

**Figure 6:**
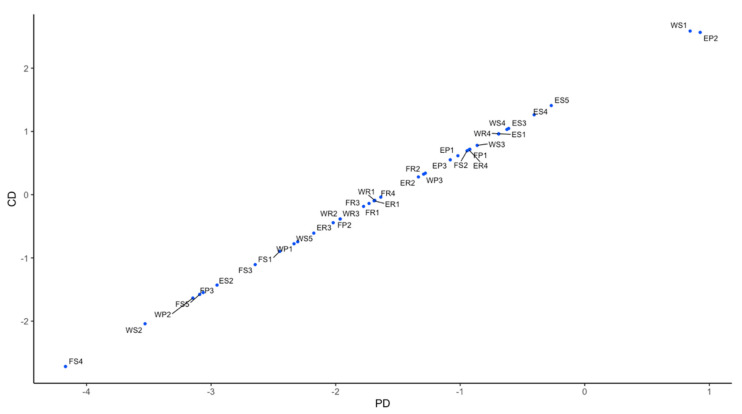
DEMATEL result–casual graph. Note: CD represents cause degree; PD represents prominence degree.

**Figure 7:**
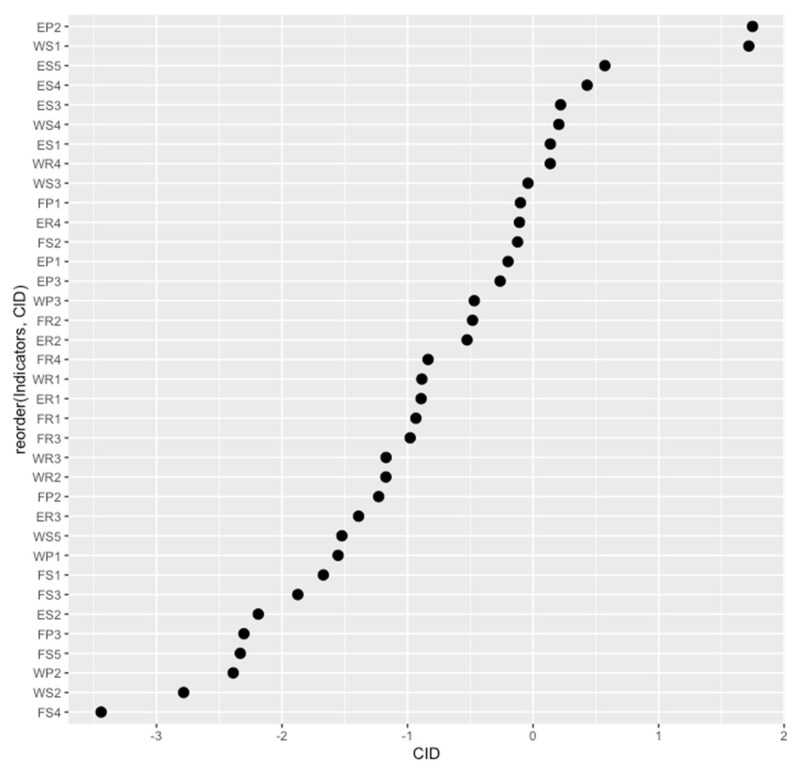
The graph of comprehensive importance degree of assessment indicators. Note: CID represents comprehensive importance degree.

When we construct the BP neural network, the number of neurons in the input layer is 36, corresponding to the 36 assessment indicators of WEF ecosystem security in Table 1. Additionally, the number of neurons in the output layer is 1, which corresponds to the symbiotic index of the WEF ecosystem. Moreover, hidden layer neural network nodes are determined according to the formula $l=\sqrt{n+k}+\alpha$, where *l* is the number of hidden layer nodes, *n* is the number of input nodes, *k* is the number of output nodes, and *α* is constant between 1 and 10. After that, letting l=7,8,…,16 for trial, we train 10 times for each assignment, and the mean-square error MSE and the coefficient of determination R^2^ are obtained after 10 trainings for each assignment, which can be the basis for comparison. After comparison, it is found that when the number of hidden layer nodes is 10, the training effect is the best and the average error is the smallest, so the number of hidden layer neurons is set to 10. Moreover, we take the highest accuracy of 500 trainings as the optimal solution. In the actual 100 trainings, the mean-square error MSE of the highest precision training is 1.35406e^−24^ and the coefficient of determination R^2^ is 1.000000. According to the DEMATEL model, we obtain the effect degree $R_{i}$, affected degree $D_{i}$, prominence degree $P_{i}$, cause degree $Q_{i}$ and comprehensive importance degree $\rho_{i}$ of 36 assessment indicators of WEF ecosystem security. Based on the results, the result–casual graph and the comprehensive importance degree graph of each assessment indicator of WEF ecosystem security are drawn, which is shown in Figure 6 and Figure 7.

It can be seen from Figure 6 that among the 36 assessment indicators of the WEF ecosystem security, cause group of influencing factor has 17 factors and the result group of influencing factor has 19 factors. The cause group’s factor is relatively stable, and its element status is difficult to improve. In addition, the result group’s factor reflects the result of the comprehensive action of the cause factors, and its element status develops and changes with the improvement of the cause factors’ status and the adjustment of the structure. In addition, nine of the cause group’s factors are related to the energy microsystem. It can be seen from this that the energy microsystem-related indicators have a greater impact on the symbiotic security of the entire WEF ecosystem.

Refer to the results, we rank the comprehensive importance degree of the assessment indicator of WEF ecosystem security and obtain the key influencing factors of the WEF ecosystem. The comprehensive importance degrees of eight influencing factors are positive. The eight factors that affect the symbiotic security of the WEF ecosystem as follows: water production modulus and water resources per capita in the water microsystem’s state dimension; daily treatment capacity of urban sewage in the water microsystem’s response dimension; energy consumption elasticity coefficient in the energy microsystem’s pressure dimension; primary energy production and energy self-sufficiency rate in the energy microsystem’s state dimension, The order of the comprehensive importance degree of each influencing factor is shown in Table 6.

### 3.5. Improvement Discussion of WEF Ecosystem Symbiotic Security

After using the two-step measurement method of symbiotic coordination to study the symbiotic relationship and the symbiotic level of the WEF ecosystem in the Yangtze River Basin, we find that there is still much room for improvement in the symbiotic development of the WEF ecosystem in the Yangtze River Basin and relevant departments need to take further effective measures to improve. Therefore, we start from the key influencing factors that affect the symbiosis and coordination of the WEF ecosystem and identify eight influencing factors that play an important role in the symbiosis and coordination of WEF. Then, based on the analysis results of key influencing factors, this article proposes strategies to promote the coordinated development of WEF from three aspects: total water resources, water pollution control, and energy demand and supply.

#### 3.5.1. Regulation Based on Water Microsystem

The Yangtze River Basin is the most abundant watershed in China, but there is still an uneven distribution. Although the total amount of water resources in the upper reaches is more abundant than in the lower reaches, the upstream water production modulus is much smaller than that in the lower reaches. In addition, the regional distribution of water resources per capita in the Yangtze River Basin is inversely proportional to the level of regional economic development. Taking 2016 as an example, the water resources per capita in the economically developed downstream regions are generally smaller than that in the upstream and midstream regions. In addition, the economically developed areas in the middle and lower reaches have the problem of pollution-induced water shortages, and sewage treatment capacity needs to be further strengthened.

By the identification of the key influencing factors of symbiosis in the WEF ecosystem, we can find that some influencing factors in the water microsystem have a great impact on the symbiosis and coordinated development of the WEF ecosystem, such as the water production modulus and water resources per capita related to the total amount of water resources, the daily treatment capacity of urban sewage-related to the water resources environment. Therefore, we will focus on the total amount of water resources and urban sewage capacity, and we will try to improve the coordination and safety of the WEF ecosystem in some areas by increasing the water production modulus, increasing the per capita water resources and improving the daily treatment capacity of urban sewage. We take the WEF ecosystem of the provinces or municipalities in the Yangtze River Basin in 2016 as an example. We select the provinces or municipalities with the lowest symbiotic index in each region of the upper, middle and lower reaches as the research object, plus the most economically developed Shanghai. After that, we increase the total amount of water resources and the daily treatment capacity of urban sewage of these four regions by ten percentage points. Then, we substitute the adjusted data into the two-step measurement method to obtain the changes in the symbiotic index of the four typical regions after improving indicators. The specific calculation results are shown in Table 7. It can be seen from the results that the symbiotic indexes of the WEF ecosystem in these four typical regions have increased significantly. Among them, Hunan has risen from a danger grade to a risk grade, and Yunnan has directly risen from a danger grade to a health grade.

In response to the inconsistency between the social and economic development and the total amount of water resources of the lower reaches of the Yangtze River Basin, relevant departments need to take certain measures to increase the per capita water resources in the region. Efforts should be made to raise the awareness of water resources protection of the people in economically developed regions, full play should be given to the important value of water resources in the river basin in the sustainable development of society, and water waste and unreasonable exploitation of water resources must be avoided. Moreover, the establishment and improvement of innovative water management systems are also very important. In terms of water environment, according to the “National Urban Sewage Treatment and Recycling Facilities Construction Plan” compiled by the National Development and Reform Commission and the Ministry of Housing and Urban–Rural Development, relevant departments should vigorously increase and upgrade the capacity of sewage treatment facilities in order to improve the sewage treatment capacity of the economically developed middle and lower reaches. For areas with a developed economy and low environmental capacity, the construction of facilities should be accelerated, the problem of uneven distribution of facilities in the region should be resolved, and stricter emission standards should be implemented.

#### 3.5.2. Regulation Based on Energy Microsystem

The Yangtze River is a huge treasure house of hydropower resources, but there is an imbalance in energy production between the east and the west. The primary energy production of most provinces or municipalities in the middle and lower reaches is far less than that of the upper reaches, which has also led to a large gap in energy self-sufficiency in the east and west. In terms of energy consumption, the developed provinces or municipalities in the lower reaches consume more energy, which also exacerbates the contradiction between energy supply and demand in the region. Moreover, market liquidity in downstream regions is relatively poor, and the process of regional market integration is relatively slow.

By the identification of the key influencing factors of symbiosis in the WEF ecosystem, we can find that some influencing factors in the energy microsystem have a great impact on the symbiosis and coordinated development of the WEF ecosystem, such as primary energy production and power generation installed capacity related to energy supply, energy consumption elasticity coefficient and energy market liquidity related to energy consumption, energy self-sufficiency rate related to energy supply and consumption. Therefore, we will focus on energy consumption and primary energy production, and we will try to improve the above indicators to ultimately promote the coordinated development of the regional WEF ecosystem. Similar to the previous section, we select Shanghai, Zhejiang, Hunan and Yunnan as the research objects. By referring to the “13th Five-Year Plan for Energy Development” issued by the National Energy Administration and the “Guiding Opinions on Energy Work” in recent years, we adjust energy consumption, primary energy production and power generation installed capacity. The specific adjustments are as follows: the annual growth rate of total energy consumption in each region in 2016 and 2017 will be controlled within 3%, the primary energy production will be increased by 2%, and the power generation installed capacity will be increased by 5.5%. Then, we substitute the adjusted data into the two-step measurement method to obtain the changes in the symbiotic index of the four typical regions after improving indicators. The specific calculation results are shown in Table 8. It can be seen from the results that the symbiotic index of WEF ecosystems in these four typical regions has increased significantly. Among them, the symbiotic grades of Zhejiang, Hunan, and Yunnan have risen by one grade.

In view of the imbalance of energy supply in the upper, middle and lower reaches of the Yangtze River, it is necessary to further implement the national regional coordinated development strategy. The energy storage and transportation capacity can be further enhanced by making full use of the Yangtze River with a huge energy-transportation channel, which can further enhance the liquidity of the energy market. In addition, the construction of inter-regional and inter-provincial power transmission channels needs to be promoted in an orderly manner. The above measures not only alleviate the problem of energy supply imbalance in the Yangtze River Basin, but also further accelerate the integration of energy markets in the downstream developed regions so as to alleviate the problem of mismatch between socio-economic development and energy supply. In addition, another good way to alleviate the pressure on energy supply in parts of the Yangtze River Basin is to accelerate the development of clean and low-carbon transition, vigorously develop non-fossil energy, do a good job in the development planning and promotion of the new energy industry, and effectively replace primary energy such as coal, oil and natural gas, so as to make up for the inadequacy of primary energy reserves in some areas and other congenital defects, thereby increasing the energy self-sufficiency rate of the region.

In general, for the current state of the WEF ecosystem in the Yangtze River Basin, it is a relatively complex system engineering to promote the coordinated and symbiotic development of the WEF ecosystem by adjusting microsystems. In the process of specific resource planning and management, it is necessary not only to seek coordinated development between water, energy and food, but also to closely integrate the social, economic and ecological conditions of the Yangtze River Basin, so as to ultimately improve the coordination and symbiosis of the WEF ecosystem.

## 4. Conclusions

This article draws on ecological symbiosis theory and developmental psychological ecological systems theory to construct a conceptual model of the WEF ecosystem from the perspective of water–energy–food symbiosis. Then, we use the two-step measurement method of symbiotic coordination to study the symbiotic relationship and the symbiotic level of the WEF ecosystem in the Yangtze River Basin. That is, the first step is to measure the WEF ecosystem’s symbiotic index to analyze and judge the symbiotic relationship and symbiotic grade of water–energy–food symbiosis, and to judge whether the WEF ecosystem symbiotic security of the provinces or municipalities in the Yangtze River Basin has reached the health threshold; The second step is to measure the symbiotic level index of the regions whose symbiotic grade of the WEF ecosystem has entered the health threshold to determine the level of water–energy–food symbiotic coordination. Finally, combined with the evaluation results of the WEF ecosystem’s symbiotic security, we provide policy recommendations for promoting the coordinated development of water, energy and food in the Yangtze River Basin by means of the identification of key factors affecting the WEF ecosystem’s symbiotic security.

We can draw the following conclusions from this research. Firstly, from the results of the WEF ecosystem symbiotic index, the average value of the WEF ecosystem symbiotic index in the Yangtze River Basin’s 11 provinces or municipalities has only reached the risk level. There is still much room for improvement in the overall symbiotic development of the WEF ecosystem in the Yangtze River Basin, and relevant departments need further optimize the symbiotic coordination of the WEF ecosystem. It is worth mentioning that the symbiosis of the WEF ecosystem in Jiangsu Province is the best, and it has been in a healthy grade for most of the 9 years. Secondly, from the results of the symbiotic level index of the regions whose WEF ecosystem’s symbiotic grade has entered the health threshold, we can find that for provinces or municipalities that have entered or have been in a healthy state, their symbiotic level is not very high. The highest symbiotic level after reaching a healthy state is not higher than 60, which occurred in Guizhou. Therefore, for provinces and municipalities where the WEF ecosystem has entered a healthy state, it is necessary to continue to optimize the collaborative management of water, energy, and food resources to further improve the coordinated development of the regional WEF ecosystem. Thirdly, from the identification of the key factors affecting symbiotic security, it can be seen that the energy microsystem-related indicators have a greater impact on the symbiotic security of the entire WEF ecosystem. In addition, the identification of key factors can provide an important reference for the planning and management of water–energy–food. The results show that resource planning and management can start from the total amount of water resources, water environment governance, energy supply and demand, so as to take targeted measures.

This article provides certain innovations and characteristics in terms of theoretical framework, judgment criteria, cause analysis, and determination of index weights. Firstly, in the theoretical framework, we apply the ecological symbiosis theory proposed by Lynn Margulis and the developmental psychological ecological systems theory proposed by U Bronfenbrenner to construct the conceptual model of the WEF ecosystem to reflect the development of each symbiotic unit that constitutes the WEF nexus nested in the symbiotic environment. Secondly, in the judgment criteria, the traditional method uses the linear weighting method to synthesize the coupling evaluation value of water–energy–food symbiosis and uses this as the only judgment standard [64]. However, for the study of the WEF ecosystem’s symbiotic security, it is not enough to measure the symbiotic index alone. Only one symbiotic index cannot judge the level of the WEF ecosystem’s symbiotic development. Therefore, this paper adopts the two-step measurement method of symbiotic coordination. Firstly, we use the Lotka–Volterra symbiotic evolution model to calculate the WEF ecosystem symbiotic index to analyze the water, energy and food symbiotic relationship and security level, and to determine whether the WEF ecosystem symbiotic security grade of each region has entered the health threshold. Secondly, we measure the symbiotic level index of each region where the WEF ecosystem is healthily symbiotic, and judge the symbiotic level of water, energy and food in the healthily symbiotic region. Thirdly, in the cause analysis, we use the modified DEMATEL model to identify the key factors affecting the symbiotic security of the WEF ecosystem. Specifically, the BP neural network method is chosen to improve the traditional DEMATEL model, thereby increasing the credibility of the identification results and analysis of key influencing factors. In addition, this improved method can also enhance the correlation between influencing factors and result evaluation factors by the process of passing error information from the output layer through the intermediate hidden layer to the input layer. Fourthly, we choose the entropy weight method and the CRITIC method to weight the evaluation indicators, which not only considers the correlation, conflict and contrast strength between the indicator information, but also considers the dispersion degree of the indicator data. Then, we use the game theory comprehensive weighting method to integrate the above two weights to obtain the comprehensive weights of each indicator, so as to minimize the deviation between the basic weights obtained by different methods and the final weights, so as to make the final weighting result tend to a more balanced state and ensure the scientific rationality of the indicator weight.

This paper uses the two-step measurement method of symbiotic coordination to measure the WEF ecosystem symbiotic index and symbiotic level index of each province or municipality in the Yangtze River Basin, so as to determine the symbiotic relationship, symbiotic grade and symbiotic coordination level of the water–energy–food symbiosis. The research focuses on the quantitative study of the symbiotic coordination of the WEF ecosystem and the analysis of the key influencing factors of the WEF ecosystem from the perspective of water–energy–food symbiosis, and there is a less specific analysis of the internal operation mechanism of WEF ecosystem. In the next step, we will further use symbiosis theory and evolutionary game theory to analyze the operation mechanism of the WEF ecosystem and explore the stability of the WEF ecosystem’s operation.

## Figures and Tables

**Table 1:** Assessment indicator system of WEF ecosystem security.

Microsystem	Dimension	Number	Indicator	Attribute
Water microsystem	Pressure (P)	WP1	Domestic water per capita	Negative
		WP2	Water resources consumption per unit GDP	Negative
		WP3	Wastewater discharge per unit GDP	Negative
	State (S)	WS1	Water production modulus	Positive
		WS2	Water production coefficient	Positive
		WS3	Water resources development and utilization rate	Negative
		WS4	Water resources per capita	Positive
		WS5	Water conservancy project storage capacity	Positive
	Response (R)	WR1	Water-saving irrigated rate	Positive
		WR2	Afforestation area	Positive
		WR3	Investment intensity of wastewater treatment	Positive
		WR4	Daily treatment capacity of urban sewage	Positive
Energy microsystem	Pressure (P)	EP1	Energy consumption intensity	Negative
		EP2	Energy consumption elasticity coefficient	Negative
		EP3	Electricity consumption per capita	Negative
	State (S)	ES1	Primary energy production	Positive
		ES2	Electricity production elasticity coefficient	Positive
		ES3	Energy self-sufficiency rate	Positive
		ES4	Energy market liquidity	Positive
		ES5	Power generation installed capacity	Positive
	Response (R)	ER1	Investment intensity of resource exploration	Positive
		ER2	Investment intensity of energy industry	Positive
		ER3	Investment intensity of energy-saving and environmental protection	Positive
		ER4	Comprehensive utilization of general industrial solid waste	Positive
Food microsystem	Pressure (P)	FP1	Non-agricultural industry output value ratio	Negative
		FP2	Food consumption per capita in rural areas	Negative
		FP3	Disaster rate of food	Negative
	State (S)	FS1	Agricultural output value index	Positive
		FS2	Food yield index	Positive
		FS3	Food production diversity index	Positive
		FS4	Volatility of total food production	Negative
		FS5	Cultivated land irrigation index	Positive
	Response (R)	FR1	Investment intensity of food and material reserves	Positive
		FR2	Fertilizer load	Positive
		FR3	Total power of agricultural machinery	Positive
		FR4	Area of cultivated land protected by dikes	Positive

**Table 2:** Symbiotic index and grade classification.

Value Range of Symbiotic Index	Symbiotic Relationship	Connotation of Symbiotic Security Grade	Symbiotic Security Grade
$-\sqrt{6}<S_{ij}\leq -1$	Mutual inhibition	Danger	1
$-1<S_{ij}\leq 1$	Unilateral promotion (inhibition)	Risk	2
$1<S_{ij}\leq \sqrt{6}$	Mutual promotion	Health	3

**Table 3:** Equilibrium coefficient and grade classification.

Grade	Coefficient	Connotation	Grade	Coefficient	Connotation
1	[0.0, 0.1)	Extremely unbalanced recession	6	[0.5, 0.6)	Barely balanced development
2	[0.1, 0.2)	Severely unbalanced recession	7	[0.6, 0.7)	Primarily balanced development
3	[0.2, 0.3)	Moderately unbalanced recession	8	[0.7, 0.8)	Intermediately balanced development
4	[0.3, 0.4)	Mildly unbalanced recession	9	[0.8, 0.9)	Well-balanced development
5	[0.4, 0.5)	On the verge of imbalance and recession	10	[0.9, 1.0]	High-quality balanced development

**Table 4:** Symbiotic index and grade of WEF ecosystem in the Yangtze River Basin.

		2008	2009	2010	2011	2012	2013	2014	2015	2016
Shanghai	Symbiotic index	−0.0335	−1.3391	−1.7047	−0.1379	0.2601	1.1960	−0.5086	−0.9771	1.0596
	Grade	2	1	1	2	2	3	2	2	3
Jiangsu	Symbiotic index	−1.0431	1.2042	1.3417	−1.4500	1.1101	1.2485	1.1693	0.9431	1.4156
	Grade	1	3	3	1	3	3	3	2	3
Zhejiang	Symbiotic index	−1.0807	1.2757	1.0505	−0.5503	0.7359	−0.4676	0.9752	0.0471	0.7953
	Grade	1	3	3	2	2	2	2	2	2
Anhui	Symbiotic index	0.8682	0.0622	0.9416	−0.9455	0.9014	−1.5138	0.7753	0.5394	0.9793
	Grade	2	2	2	2	2	1	2	2	2
Jiangxi	Symbiotic index	0.1284	−1.0914	−0.9987	−0.2474	1.4304	1.0007	0.9603	−0.0223	0.5389
	Grade	2	1	2	2	3	3	2	2	2
Hubei	Symbiotic index	1.4202	1.0503	−1.6715	0.5860	−1.0673	0.1578	−1.3513	−1.2204	0.4787
	Grade	3	3	1	2	1	2	1	1	2
Hunan	Symbiotic index	0.3166	1.5451	0.6240	0.5919	0.9838	0.0805	−0.4819	−1.4435	−0.8866
	Grade	2	3	2	2	2	2	2	1	2
Chongqing	Symbiotic index	1.0583	−1.6340	−0.1418	−0.0444	0.7741	−0.3131	0.3682	−0.5177	1.2936
	Grade	3	1	2	2	2	2	2	2	3
Sichuan	Symbiotic index	−0.1677	−1.3058	1.2738	0.8232	0.7599	−0.9575	−1.2029	−1.0121	0.4132
	Grade	2	1	3	2	2	2	1	1	2
Guangzhou	Symbiotic index	−1.2637	0.8118	0.8483	0.8721	1.2049	0.2842	1.2654	0.9319	0.6487
	Grade	1	2	2	2	3	2	3	2	2
Yunnan	Symbiotic index	−0.4988	−0.8032	1.1121	0.1339	0.7955	−1.1142	−1.1884	−1.6984	−1.0001
	Grade	2	2	3	2	2	1	1	1	1

**Table 5:** Symbiotic level index of WEF ecosystem in the Yangtze River Basin.

		2008	2009	2010	2011	2012	2013	2014	2015	2016
Shanghai	$\tau_{i}$	—	—	—	—	—	—	—	—	0.6863
	Grade	—	—	—	—	—	—	—	—	7
	$G_{i}$	—	—	—	—	—	—	—	—	28.4726
Jiangsu	$\tau_{i}$	—	0.5062	0.4978	—	0.6276	—	0.7150	—	0.7241
	Grade	—	6	5	—	7	—	8	—	8
	$G_{i}$	—	7.9373	8.4362	—	9.7675	—	12.0592	—	14.4395
Zhejiang	$\tau_{i}$	—	0.4547	0.4085	—	—	—	—	—	—
	Grade	—	5	5	—	—	—	—	—	—
	$G_{i}$	—	10.2297	8.6375	—	—	—	—	—	—
Anhui	$\tau_{i}$	—	—	—	—	—	—	—	—	—
	Grade	—	—	—	—	—	—	—	—	—
	$G_{i}$	—	—	—	—	—	—	—	—	—
Jiangxi	$\tau_{i}$	—	—	—	—	0.7086	0.6338	—	—	—
	Grade	—	—	—	—	8	7	—	—	—
	$G_{i}$	—	—	—	—	46.5109	32.9509	—	—	—
Hubei	$\tau_{i}$	0.6891	0.6431	—	—	—	—	—	—	—
	Grade	7	7	—	—	—	—	—	—	—
	$G_{i}$	28.4717	22.7153	—	—	—	—	—	—	—
Hunan	$\tau_{i}$	—	0.8537	—	—	—	—	—	—	—
	Grade	—	9	—	—	—	—	—	—	—
	$G_{i}$	—	35.6857	—	—	—	—	—	—	—
Chongqing	$\tau_{i}$	0.5434	—	—	—	—	—	—	—	0.8527
	Grade	6	—	—	—	—	—	—	—	9
	$G_{i}$	36.5556	—	—	—	—	—	—	—	51.1993
Sichuan	$\tau_{i}$	—	—	0.5815	—	—	—	—	—	—
	Grade	—	—	6	—	—	—	—	—	—
	$G_{i}$	—	—	22.1169	—	—	—	—	—	—
Guizhou	$\tau_{i}$	—	—	—	—	0.4201	—	0.4594	—	—
	Grade	—	—	—	—	5	—	5	—	—
	$G_{i}$	—	—	—	—	55.2523	—	58.9536	—	—
Yunnan	$\tau_{i}$	—	—	0.4812	—	—	—	—	—	—
	Grade	—	—	5	—	—	—	—	—	—
	$G_{i}$	—	—	41.5607	—	—	—	—	—	—

**Table 6:** The key influencing factors of the WEF ecosystem.

Ranking	Number	Key Influencing Factors	$\rho_{i}$
1	EP2	Energy consumption elasticity coefficient	1.7484
2	WS1	Water production modulus	1.7189
3	ES5	Power generation installed capacity	0.5717
4	ES4	Energy market liquidity	0.4305
5	ES3	Energy self-sufficiency rate	0.2195
6	WS4	Water resources per capita	0.2035
7	ES1	Primary energy production	0.1377
8	WR4	Daily treatment capacity of urban sewage	0.1370

**Table 7:** Results of WEF ecosystem control based on water microsystem.

Regions	Symbiotic Index before Improvement	Symbiotic Index after Improvement
Shanghai	1.0596	1.3500
Zhejiang	0.7953	0.9956
Hunan	−0.8866	0.6526
Yunnan	−1.0001	0.8136

**Table 8:** Results of WEF ecosystem control based on energy microsystem.

Regions	Symbiotic Index before Improvement	Symbiotic Index after Improvement
Shanghai	1.0596	1.2110
Zhejiang	0.7953	1.0121
Hunan	−0.8866	0.7429
Yunnan	−1.0001	−0.2520

## Data Availability

Not applicable.
